# Systematic Review for Risks of Pressure Injury and Prediction Models Using Machine Learning Algorithms

**DOI:** 10.3390/diagnostics13172739

**Published:** 2023-08-23

**Authors:** Eba’a Dasan Barghouthi, Amani Yousef Owda, Mohammad Asia, Majdi Owda

**Affiliations:** 1 Health Sciences Department, Arab American University, Ramallah P600, Palestine; e.dasan@student.aaup.edu (E.D.B.); mohammad.asia@aaup.edu (M.A.); 2 Department of Natural Engineering and Technology Sciences, Arab American University, Ramallah P600, Palestine; 3 Faculty of Data Science, Arab American University, Ramallah P600, Palestine; majdi.owda@aaup.edu

**Keywords:** pressure injury, prediction model, nursing care, machine learning, inpatient

## Abstract

Pressure injuries are increasing worldwide, and there has been no significant improvement in preventing them. This study is aimed at reviewing and evaluating the studies related to the prediction model to identify the risks of pressure injuries in adult hospitalized patients using machine learning algorithms. In addition, it provides evidence that the prediction models identified the risks of pressure injuries earlier. The systematic review has been utilized to review the articles that discussed constructing a prediction model of pressure injuries using machine learning in hospitalized adult patients. The search was conducted in the databases Cumulative Index to Nursing and Allied Health Literature (CINAHIL), PubMed, Science Direct, the Institute of Electrical and Electronics Engineers (IEEE), Cochrane, and Google Scholar. The inclusion criteria included studies constructing a prediction model for adult hospitalized patients. Twenty-seven articles were included in the study. The defects in the current method of identifying risks of pressure injury led health scientists and nursing leaders to look for a new methodology that helps identify all risk factors and predict pressure injury earlier, before the skin changes or harms the patients. The paper critically analyzes the current prediction models and guides future directions and motivations.

## 1. Introduction

“A pressure injury (PI) can range from skin erythema to injured muscle and underlying bone, depending on the impacted tissue layer’s size and degree” [1]. It is also known as a pressure ulcer, decubitus ulcer, or bedsore. Depending on the impacted tissue layer’s size and degree, it can range from skin erythema to injured muscle and underlying bone [1].

A pressure injury is a significant issue in providing healthcare and maintaining patient safety, with a global prevalence of 12.8% and hospital-acquired pressure injuries (HAPI) of 8.4% [2]. Moreover, 2.5 million patients in the United States of America (USA) develop pressure injuries annually in acute care settings [3]. 95% of pressure injuries are preventable, and the expenditures for measures to prevent pressure injuries are lower than the treatment expenditures. This led to pressure injuries being a vital quality indicator in healthcare organizations [4].

Pressure injuries impact patients’ quality of life, morbidity, and mortality and increase the burden on healthcare expenditures [1]. In addition to the harm that affects the patient who seeks help and care, it affects patients’ safety negatively and extends the hospitalization period [5].

Many factors are associated with PIs, like age, gender, hospital length of stay, limited mobility, disease severity, skin condition, medications, anesthesia, type of surgery, diagnosis, and nursing workload [4,6,7]. In addition, patients who suffer from PI complain of pain, recurrent infections, hospital admissions, ongoing use of antibiotics, prolonged hospital stays, and psychosocial impact [8,9].

**Figure 1:**
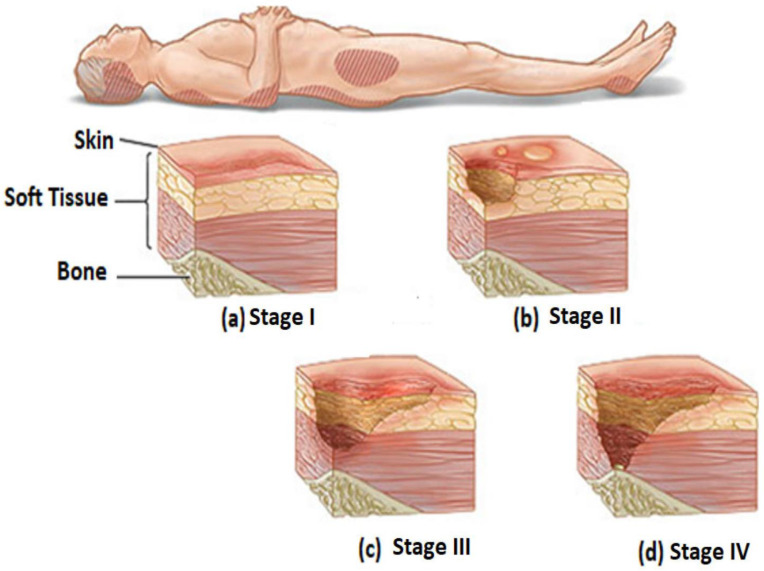
Stages of pressure sores [18] classify into four main stages: stage I, where the pressure injury affects tissue perfusion or circulatory or skin erythema (**a**); stage II, where the pressure injury affects the thickness of the tissue and causes loss of dermis (**b**); stage III, where the pressure injury causes necrosis to the tissue or loss of the deep layer of tissue (**c**); and stage IV, where the pressure injury affects the full thickness of the tissue and destroys the tissue layer and subcutaneous fat (**d**).

Stages of pressure injury range from stage I to IV (as illustrated in Figure 1); these stages are classified based on the harm to the affected body area or the degree of tissue layer that is affected; stage “I” is defined when the pressure injury affect the tissue perfusion or circulatory or skin erythema, stage “II” when the pressure injury affect the thickness of the tissue and cause loss of dermis, stage “III” is defined that the necrosis to the tissue or loss of the deep layer of tissue, and finally, stage “IV” it means that the pressure injury affects full thickness of tissue and cause destruction of the tissue layer and subcutaneous fat be visible [10], as shown in Figure 1.

Many studies identified the risk factors of pressure injury and identified the interventions and evidence to treat the patient who developed pressure injury. Most of these studies acknowledged the complexity of pressure injury, which mainly referred to the multiple factors accompanying the affected patients and the treatment environment. In other words, hospital-acquired pressure injury results from the dynamics of nonlinear contributing factors in the care process and patient interaction [11].

A hospital-acquired pressure injury is considered one of the specific nursing indicators, and the cornerstone to protecting the patient from any complications is to identify the risks of PI earlier and implement the prevention measures [12].

Risk assessment tools are widely used to identify patients at risk of developing a PI. These traditional tools (Braden, Norton, and Waterlow Scales) need to identify valuable risk factors, such as age and hemoglobin [13]. Those tools rely on subjective measures such as the skin’s condition and the friction range between the skin and beds [4].

Using machine learning (ML) assists in predicting the risk of PI by utilizing vast amounts of data embedded in the electronic medical record, and it may also help nurses identify pressure injuries earlier and promote patient safety. However, this approach is still in its early stages and will be investigated and tested in PIs [14].

It is worth mentioning that this paper reviews the pressure injury risks and predictive risk factors generated from the prediction models; the other review studies [15,16,17] did not investigate the predictive risk factors generated from the prediction models of pressure injury. The study conducted by Dweekat, Lam, and McGrath [15] presents findings from 30 studies using prediction models of pressure injury; 29 utilized machine learning, and one used deep learning. The algorithms were DT, LR, SVM, LD, RF, MLP, and KNN. Although the paper concluded that all studies discussed risk factors that may predict pressure injuries. However, none of the studies investigated or tracked the impact of changing the status of the risk factors during the hospitalization period. The study conducted by Jiang et al. in [16] included 32 studies; only 11 studies related to the ML to detect pressure injury earlier. This study concludes that the decision tree algorithm was the dominant approach. The study conducted by Qu et al. in [17] shows findings from 25 studies. It indicates five ML models, i.e., DT, RF, SVM, NN, and LR, and according to this study, the RF was the best algorithm to predict pressure injury.

This paper presents an Introduction in Section 1; Materials and Methods (research design and protocols, search strategy, and inclusion and exclusion criteria) in Section 2; Results (the risk factors and biomarkers, predictive risk factors, the prediction models of pressure injury with their features, and summaries of the studies that discussed the prediction models) in Section 3; and discussion of the findings in Section 4. Finally, conclusions and motivations for future directions are in Section 5.

## 2. Materials and Methods

The systematic review has been utilized to review the articles that discussed constructing a prediction model of pressure injuries using machine learning in hospitalized adult patients. Machine learning assists in predicting the risk of pressure injury by utilizing vast amounts of data embedded in the electronic medical record, and it may also help nurses identify pressure injuries earlier and promote patient safety. The methodology used in this paper is divided into five sections, namely: (1) research design and protocols; (2) search strategy; (3) study selection method; (4) inclusion and exclusion criteria; and (5) quality assessment.

### 2.1. Research Design and Protocols

A systematic review was conducted on pressure injury risk factors and prediction models and reported according to Preferred Reporting Items for Systematic Reviews and Meta-Analyses (PRISMA) guidelines [19].

### 2.2. Search Strategy

We conducted a systematic review of five different health science databases used in this research: Cumulative Index to Nursing and Allied Health Literature (CINAHIL), PubMed, Science Direct, Institute of Electrical and Electronics Engineers (IEEE), Cochrane, and Google Scholar search.

The keywords utilized in this research were pressure ulcer, pressure injury, pressure sore, decubitus ulcer, decubitus sore, bedsore, machine learning, and adult hospitalized patients. Two Boolean operators were used (OR and AND), and the search period included the studies relevant to the topic and research purpose between 2017 and 2023.

### 2.3. Study Selection Method

Two independent researchers used the eligibility criteria to evaluate the titles and abstracts. The entire texts of all possible publications were then obtained, and they were independently examined. Any disagreement regarding the study’s inclusion was handled or discussed with a third researcher.

### 2.4. Inclusion and Exclusion Criteria

This review includes the studies that met the inclusion criteria for this search, including those using machine learning to predict pressure injuries in inpatients and adult patients. The language of the literature is English. In addition, the study excluded patients younger than 14 years, patients with pressure injuries acquired from outside the hospital, and papers that did not recruit machine learning algorithms to predict pressure injuries.

### 2.5. Quality Assessment

The quality assessment was performed according to the Joanna Briggs Institute’s (JBI) critical appraisal checklist by two independent reviewers (E.D.B. and A.Y.O.) to assess the risk of bias in the included studies [20]. Any disagreement regarding the judge was handled or discussed with a third researcher. The JBI checklist consists of 11 items; each item was scored by yes, no, unclear, and not applicable, and the overall score is assessed for each study and sorted by risk of bias (high, moderate, low) as per the JBI checklist. The score was categorized as high risk if the total of each item was less than 50, moderate if it was between 51 and 80, and low if it was between 81 and 100.

## 3. Results

The existing literature has focused on different aspects of pressure injury and the prediction model of pressure injury. Overall, 494 studies appeared in the literature search (485 from the databases and 8 from the Google search), and 19 were removed due to duplicate records. From those, 426 studies were removed due to the fact that the articles were not related to the prediction model of pressure injury, and 48 studies were reviewed to assess the inclusion criteria; out of the 48 studies, two were excluded due to the lack of reports available, and only 46 articles were screened to assess if the studies matched the inclusion criteria. Of the 46 studies, 2 were not developing models but only protocols for review; 7 were for pediatric patients; and 10 were for community-acquired pressure injuries. Finally, 27 studies were included in the systematic review due to the availability of free full texts and the complete matching of the inclusion criteria.

**Figure 2:**
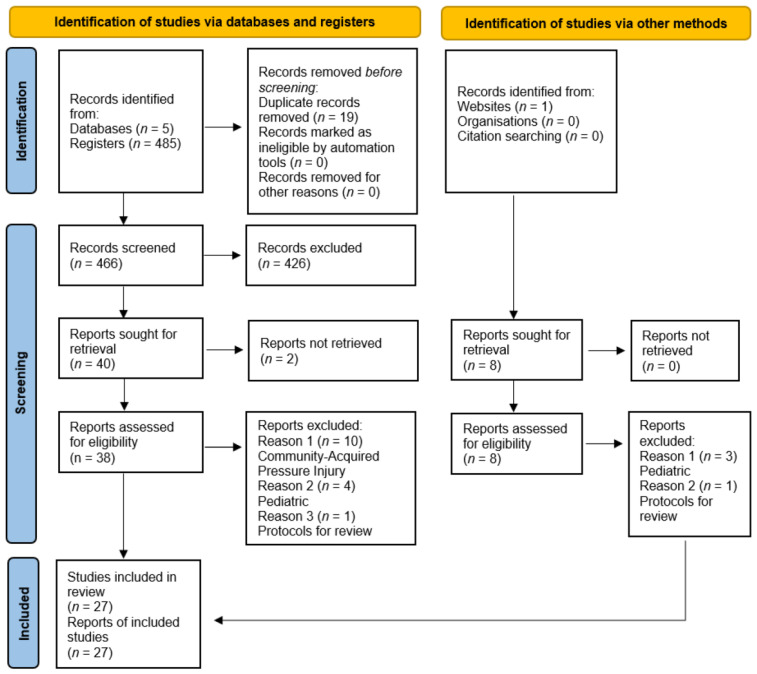
PRISMA for the systematic review conducted in this research [19].

The findings of searching and the screening method were explained in the PRISMA for systematic review, as shown in Figure 2, and the PRISMA was utilized to improve the accuracy of reviewing studies and be more helpful.

### 3.1. Characteristics of Included Studies

The utilization of machine learning to predict pressure injuries was discussed in many studies described in Table 1, and they concluded that machine learning had a promising future in detecting pressure injuries. Table 1 summarizes the different study designs and sample sizes of the studies included in this review that discussed the prediction models for pressure injuries.

Those studies used different designs, such as prospective (five studies), retrospective (sixteen studies), experimental (one study), case study (one study), prospective and retrospective (one study), and systematic review and meta-analysis (three studies). Also, those studies utilized various types of data sources, such as databases for electronic medical records, patient observation, and reviewing medical records. The dataset size of the patient medical records included in those studies ranged from 149 to 237,397.

The sample size and datasets utilized in the reviewed studies ranged from small to large data sets; this variation is considered one of the challenges in machine learning. Furthermore, the data imbalance problems affect the proposed models’ training, as reported in [21]. The reviewed studies utilized different approaches in the data balances (Random Oversampling, Synthetic Minority Oversampling, and Undersampling), as illustrated in Table 1. Most of the reviewed studies included in this paper used Random Oversampling at 34%, Synthetic Minority Oversampling at 14%, and Undersampling at 7%. Finally, about 45% have not reported the balance method or said it is not applicable.

**Table 1:** Characteristics of the included studies.

Reference	Design	Sample Size	Method Balancing
[22]	Cohort Retrospective	269	Not Reported
[23]	Cohort Prospective	648	Not Reported
[15]	Systematic Reviews	168–188,512	Not Applicable
[17]	Systematic Reviews	237,397	Not Applicable
[24]	Cohort Retrospective	486	Not Reported
[25]	Cohort Prospective	12,654	Random Oversampling
[26]	Cohort Retrospective	50,851	Synthetic Minority Oversampling and Random Oversampling
[14]	Cohort Retrospective	6376	Random Oversampling
[4]	Experimental Design	11,838	Under Sampling and Random Oversampling
[16]	Systematic Reviews and Meta	125,213	Not Applicable
[27]	Cohort Retrospective	100,355	Not Reported
[28]	Cohort Retrospective	4652	Random Oversampling
[29]	Cohort Retrospective	618	Random Oversampling
[30]	Cohort Retrospective	75,353	Random Oversampling
[31]	Case-Control	2341	Under Sampling
[32]	Cohort Prospective	13,254	Not Reported
[33]	Cohort Prospective	194	Not Reported
[34]	Cohort Retrospective	18,019	Not Reported
[35]	Cohort Prospective	149	Not Reported
[36]	Cohort Retrospective	15,310	Random Oversampling
[37]	Cohort Retrospective	9644	Not Reported
[38]	Cohort Retrospective	5101	Synthetic Minority Oversampling
[39]	Cohort Retrospective	18,943	Synthetic Minority Oversampling
[13]	Cohort Retrospective and Prospective	6694	Synthetic Minority Oversampling
[40]	Cohort Retrospective	149,006	Random Oversampling
[41]	Cohort Retrospective	6742	Random Oversampling
[42]	Cohort Retrospective	206,540	Not Reported

Different studies discussed the use of machine learning in constructing a prediction model for pressure injury; 27 studies were reviewed in the literature in terms of using machine learning to predict pressure injury [4,9,11,13,14,17,20,21,22,23,26,27,29,30,31,32,34,35,36,43]. Those studies focused on different aspects of pressure injury and the department or specialty when the patient developed pressure injury, as Ji-Yu et al. [32] developed a prediction model for patients undergoing cardiovascular operations, and the model predicts pressure injury based on the clinical data; Walther et al. [36] studied the power of risk factors related to pressure injury by utilizing machine learning technology; the data were collected retrospectively from 2014 to 2018. Most studies were conducted to track the intensive care unit patients (14 out of 27).

Most of the data sets utilized in the reviewed papers are generated from the electronic medical records conducted in that hospital (20 studies), followed by a national database (3 studies), an international database (1 study), and systematic reviews (3 studies).

The sampling of the reviewed studies included in the paper is free of limitations related to the ethnic background, socioeconomic status, and gender of the patients that participated in the studies. For the age group, most of the studies determined the adult age for the participants at 70% of the included studies, followed by free of limitations (adult and pediatric) at 19%, not reported in the manuscript at 11%, and elderly patients above 65 years at 4%. Finally, most reviewed papers determined the pressure injury rate for the patients admitted to the intensive care units to be 52%.

### 3.2. Risk of Bias Assessment

**Figure 3:**
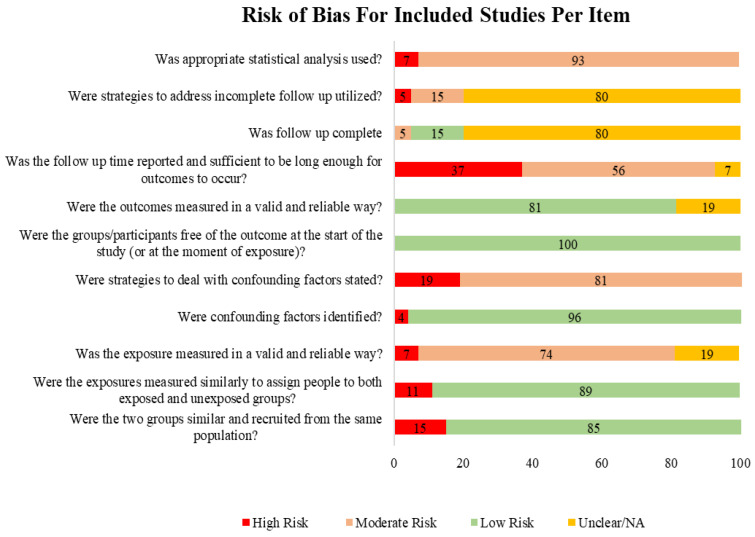
Risk of bias according to the items of the JBI checklist. Categorized into High, Moderate, Low, and Unclear or Not applicable (NA). The items of the JBI (11) and rating through (Yes, No, Unclear, and NA). High Risk (Less than 50%, Moderate 51–80%, and Low 81–100%). Color coding: Red (High Risk), Orange (Moderate Risk), Green (Low Risk), and Yellow (Unclear/Not Applicable).

One item in the JBI checklist was judged low risk; others ranged from high to low risk. However, they were either not included in the manuscripts or had inadequate information, and there were certain items where the risk of bias was unclear. Figure 3 illustrates the risk of bias across all items on the JBI checklist.

### 3.3. Risk Factors and Biomarkers of Pressure Injury

The studies conducted to identify the risk factors and biomarkers of pressure injury were in six articles [8,14,43,44,45,46] summarized in Table 2. The incidence and prevalence of pressure injuries were discussed worldwide in many studies, such as [47,48,49]. A study was conducted in 2019 by Qaddumi et al. [48] to assess the incidence rate of pressure injury and its related variables through a prospective design for 140 admitted adult patients to the ICU and assessing them by the Braden scale to identify the risk of pressure injuries during a stay at the ICU. The findings of the study were that 30% of patients developed pressure injuries; for other variables, the frequency of bed repositioning and folly’s catheter are not significant but protective factors for pressure injury in ICU patients. The limitations faced in this study are the small sample size and the data collection depending on the nurses in those hospitals.

Various risk factors affect pressure injuries, some of which are predictor variables [43]. Those factors may include but are not limited to age, gender, body mass index, length of stay, medications, vital signs, anesthesia, the Braden scale, the Braden subscale (sensory perception, moisture, activity, mobility, nutrition, and friction and shear), and diagnoses such as cancer, cardiovascular disease, diabetes mellitus, renal failure, and respiratory disease [14,43]. Table 2 summarizes the risk factors and biomarkers identified in those articles.

The visual skin assessment (VSA) to predict pressure injury relies on assessment tools that cannot be reliable prediction methods [8], and these methods are limited and problematic because pressure injury develops from the deep tissue; it cannot be noticed until it reaches the skin layer [44].

Objective measures to predict pressure injury called biomarkers, defined as the normal reaction to physiological skin irritation [8], have significant potential to identify the risks of pressure injury through identifying inflammation activated by the inflammation biomarkers such as keratinocytes before the skin changes and skin injury [46].

The research of Schwartz et al. [45] identifies the correlation between pressure injury and biomarkers for the patient after spinal cord injury. It was shown that the circulatory biomarkers and muscle-based biomarkers could identify patients with a high risk for recurrent pressure injury, which found that muscle quality is an effective biomarker and the biomarkers of Fatty Acid-Binding Protein (FABP4) circulator inflammatory factor had a significant level for recurrent pressure injury after spinal cord injury.

The potential of biomarkers for the early detection of pressure injuries was assessed by [44,46]. Those studies focus on inflammatory biomarkers. The first study [44] investigates IL-1a (total protein) with sub-epidermal moisture (SEM) and finds a weak correlation between Interleukin-1 Alpha (IL-1a) and SEM [44]. In contrast, the other research [46] explores the creatine kinase (CK), heart-type fatty acid binding protein (H-FAB), and myoglobin (Mb) for the control and spinal cord injury (SCI) groups. It concludes that the two groups (control and SCI) have a positive relationship between the CK and heart-type fatty acid binding protein (H-FAB) and between the Mb and H-FAB. Only H-Fab and CRP had higher concentrations than other subjects [46].

A systematic review study conducted by Wang et al. in [8] discusses the biomarkers that may detect pressure injury and the role of the biomarkers in early detection, which involved Alb, Waterlow score, hemoglobin (Hb), C-Reactive Protein (CRP), age, gender, H-FABP, granulocyte-macrophage colony-stimulating factor (GM-CSF), IL-15, TNF-α, and Interferons—Alpha (IFN-a) in urine). The study [8] concludes that the combination of gender, age, Hb, albumin (Alb), and CRP is the most significant biomarker [8].

### 3.4. Predictive Risk Factors and Biomarkers of Pressure Injury

The pressure injury risk factors are vast, and the staff cannot predict all cases due to the unique patient differences [30]. In addition, the pressure injury harms the patients, affecting outcomes and treatment plans, which may cause significant harm in severe cases before the staff detects the pressure injury [14]. So, the prediction model of pressure injury was studied to assess the applicability and benefits of identifying the pressure injury earlier and alarming the system with the risk of pressure injury for the admitted patients due to certain factors and biomarkers [30].

According to Sir William Osler [50], “Medicine is a science of uncertainty and an art of probability”. This is the evolution of a new approach to medicine, indicating the importance of machine learning in the healthcare industry and forming the promising future of artificial intelligence [50].

The utilization of machine learning to construct a prediction model for pressure injury differs in the predictive risk factors that resulted from the prediction models; the subsequent studies present the predictive risk factors that resulted from the prediction models.

The Xu et al. study [29] found that the predictive risk factors were the reason for admission, clinical laboratory results, patients’ demographics, medical history, and Braden scale. Another study by Shui et al. [31] found that the predictive risk factors were patients’ demographics, medications, diagnosis, ventilation, and incidence of HAPI.

Also, the Cramer et al. study [26] found that the predictive risk factors were age, gender, weight, mean arterial pressure, consciousness, medications, diagnoses, laboratory, and incidence of PI. Another study by Alderden et al. [14] found that the predictive risk factors were vasopressor, temperature, blood pressure, sedation, severity of illness, oxygenation, and confusion level.

Moreover, a study by Tang et al. [23] found that the predictive risk factors were age, gender, weight, body mass index, albumin, Hb, and comorbidities. Another study by Choi et al. [33] found that the predictive risk factors were oral mucosal, endotracheal tube (ETT), vasopressor, albumin, hematocrit (HCT), and steroids. A study by Anderson et al. [39] examined age, gender, diagnoses, length of stay, comorbidities, the severity of illness, and the Braden scale.

Furthermore, a study by Nakagami et al. [30] found that the predictive risk factors were age, gender, diagnoses, diet, pain, paralysis, level of consciousness, skin condition, comorbidities, the severity of illness, and department type. Another study by Sun et al. [24] found that the predictive risk factors were age, gender, diagnosis, cancer, anti-cancer therapy, Waterlow score, laboratory results, medications, length of stay, mechanical ventilation, acute physiology and chronic health evaluation (APACHE) II score, and blood purification. A study by Ladios-Martin [13] found that the predictive risk factors were gender, age, place of birth, hospital, diagnosis, and APACHE II score.

Deschepper et al. [32] found that the predictive risk factors were age, gender, diagnosis, Braden score, body mass index (BMI), heart rate, mean arterial pressure, temperature, laboratory results, and immunocompromised status. Table 3 summarizes the predictive risk factors identified in those articles.

The common risk factors investigated in most of the studies were diseases and comorbidities, laboratory results, Braden scale, use of medications, age, vital signs, gender, body mass index, length of stay, duration of surgery, and critical conditions, such as the following factors that correlate with pressure injuries; age > 74 years, female ASA ≥ 3, BMI < 23, Braden score, anemia, respiratory disease, and HTN were studied by Aloweni et al. [22]; High FBS, vasoactive drugs, and the duration of surgeries were studied by Tang, Li, and Xu [23]; the critical condition and high Barden were studied by Sun et al. [24]; length of the patient’s stay was studied by Šín et al. [28]; prolonged length of stay in the ICU, DM, male gender, BMI, and maximum lactate were studied by Deschepper et al. [32]; mechanical ventilation, anesthesia, and age were studied by Walther et al. [40].

### 3.5. Machine Learning Prediction Model of Pressure Injury

This paper reviewed 27 studies, which included 24 studies that construct prediction models of pressure injury [13,14,16,17,20,21,22,24,25,26,27,28,29,30,31,32,33,34,35,36,37,38], and 3 out of those 27 were systematic reviews.

Those studies recruit many types of machine learning algorithms; some of those studies recruit one kind of algorithm, and some of the studies recruit more than one type of algorithm, as described in Table 4; these algorithms were Neural Network (NN), Decision Tree (DT), Regression Tree (RT), Linear Discriminants (LD), Super Vector Machine (SVM), Random Forest (RF), Logistic Regression (LR), K-Nearest Neighbor (KNN), Gradient Boosting Machine (GBM), Explainable Boosting Machine (EBM), Bayesian Networks (BN), Extreme Gradient Boosting (XGBoost), Gradient Boosting (GB), and Bayesian Additive Regression Trees (BART).

Different approaches evaluated the machine learning prediction models of pressure injury, and most of those models utilized the Area Under the Curve (AUC) in addition to the other performance metrics such as accuracy, sensitivity, specificity, precision, and recall. Finally, not all studies report all the attributes of the performance metrics.

## 4. Discussion

This section will discuss the results obtained from all reviewed papers (risk factors and biomarkers of pressure injury, characteristics of included studies, and prediction models of pressure injury) and the research implications, limitations, and recommendations for future directions.

### 4.1. Discussion of Results

We examined 494 journal articles and picked 27 that offered information about the machine learning prediction model of pressure injury utilized in hospital settings to identify the pressure injury earlier in our systematic review. Furthermore, the studies discussed using machine learning to predict pressure injuries in adult inpatients. In total, 27 articles were included in the review, and the following themes were considered: risk factors and biomarkers of pressure injury; characteristics of the included studies; and prediction models of pressure injury.

We notice that no studies look for all risk factors and biomarkers of pressure injury. The reviewed studies utilized different design approaches to predict pressure injuries. In addition, the prediction model of pressure injury provides clear evidence that machine learning algorithms will assist healthcare providers by identifying the pressure injury earlier and with a high accuracy rate. We offer a complete overview of the reviewed articles on the prediction model of pressure injury utilized in hospital settings.

Utilizing the balance methods will improve the prediction model results [51]. The studies conducted in [28,36] showed excellent performance of the proposed predictive models due to the use of balancing methods. Moreover, utilizing approaches to overfitting in the development of new models is highly recommended, especially for models with low performance.

#### 4.1.1. Characteristics of Included Studies

In this review, we focus on the studies that use the machine learning prediction model of pressure injury and those that utilize different research designs to predict pressure injury. Most of those studies utilized retrospective studies that enabled the researchers to obtain the data from data warehouses (14 out of 27 studies). It is worth mentioning that there is one study that utilized the experimental design to construct the prediction model of pressure injury. This approach matches a content analysis study by Kamiri and Mariga [52] to discuss the research methodology in machine learning, which revealed that all studies included in the analysis used machine learning in experimental designs.

#### 4.1.2. Risk Factors and Biomarkers of Pressure Injury

Pressure injury (also called pressure ulcers) has many risk factors and biomarkers that may affect patients and potentially affect the incidence of pressure injury. These factors are not standardized for all patient categories. The factors that affect pressure injury identified in the prediction model studies included in this review and those identified as predictive factors. This means that those factors are significant and correlated with a pressure injury.

The most prevalent factors among the studies were age, gender, body mass index, length of stay, medications, vital signs, anesthesia, Braden scale, Braden subscale (sensory perception, moisture, activity, mobility, nutrition, and friction and shear), diagnoses include cancer, cardiovascular, diabetes mellitus, renal failure, respiratory, and diagnostic tests include FABP4, IMAT, IL-1α, SEM, CK, H-FAB, Mb, Alb, Hb, CRP, IL-15, TNF-α, IFN-α, and GM-CSF [8,14,39,40,41,42]. These findings are relevant to risk factors identified in other studies conducted to assess the risk factors of pressure injury [53,54].

#### 4.1.3. Machine Learning Prediction Models of Pressure Injury

The main objective of this review is to identify the studies that utilized machine learning in the prediction model of pressure injury and to summarize the approaches and algorithms used in these studies. In addition to summarizing those models’ performance metrics and evaluation methods and the results obtained from the prediction models.

All studies rely on data available in the data warehouses of electronic medical records. Furthermore, most studies do not describe the preprocessing of the data or data cleaning. Most studies did not mention the selection process of algorithms, and LR was the most frequent machine learning algorithm, followed by RF, SVM, NN, and DT. These results match the study by Kamiri and Mariga [52].

Moreover, all studies concluded that the prediction model successfully predicts pressure injury in the ICU, CCU, open wards, and hospital settings for admitted adult patients based on the clinical data in the electronic medical record [13,14,16,17,20,21,22,24,25,26,27,28,29,30,31,32,33,34,35,36,37,38]. A pressure injury is considered a significant issue in the healthcare industry and affects patient safety and quality of care. So, the prediction model provides a promising tool for healthcare providers, mainly nurses, to predict pressure injuries earlier.

The hospital management needs to provide their hospitals with models to assist their staff with prediction models to detect pressure injuries earlier. The prevalence of pressure injuries in hospitals is still high, and the health system and policymakers may need to recruit new methods that identify the risks of pressure injuries. Furthermore, the prediction model of pressure injury needs to be implemented on a different level and provided to healthcare facilities with this model that helps the healthcare providers identify the risk of pressure injuries, or the patient may develop pressure injuries during the hospital stay.

### 4.2. Research Implication

The prevalence of pressure injuries in hospitals is still high, and the health system and policymakers may need to recruit new methods that identify the risks of pressure injuries. Furthermore, the prediction model of pressure injury needs to be implemented on a different level and provided to healthcare facilities with this model that helps the healthcare providers identify the risk of pressure injuries, or the patient may develop pressure injuries during the hospital stay.

### 4.3. Limitations of the Research

The systematic review includes articles from the most reputable five databases and a Google Scholar search, which may consist of only some relevant articles from all databases. Also, the research reviewed the articles in English only.

### 4.4. Recommendation

The findings of this review suggest that nurses, physicians, physiotherapists, and dieticians may benefit from models that predict pressure injuries in hospital settings; these models can provide a valid tool in addition to implementing evidence-based practices that will mitigate and prevent pressure injuries. The hospital management needs to provide their hospitals with models to assist their staff with prediction models to detect pressure injuries earlier.

Finally, the literature review from the previous work on the prediction model of pressure injury shows that the prediction model predicts which patients may develop pressure injury based on the risk factors that belong to the patients but does not predict when the patients may acquire the pressure injury. Furthermore, the prediction model recommends tracking changes in the patient’s status, condition, or biomarkers resulting in pressure injury to identify whose patients may acquire the pressure injury during hospitalization. Also, one of the gaps in the previous works is that no one has studied, investigated, or mentioned accreditation status as a variable or feature in the prediction model developed in those studies. The accreditation status means an accreditation body or agency acknowledges the hospital’s implementation of the accreditation standards [55].

## 5. Conclusions

The number of ML approaches utilized in the reviewed studies was 21; the top five were logistic regression, random forest, decision tree, support vector machine, and neural network. Logistic regression was the dominant approach with 28% of all used models, followed by random forest with 20%, decision tree with 11%, support vector machine with 9%, and neural network with 7%. This means that 75% of the reviewed studies used the top five models, whereas 25% used other ML models. It is worth mentioning that, according to the findings, logistic regression and random forest were the best models to predict pressure injury. The common risk factors were investigated in most of the studies, and we found that those factors are diseases and comorbidities (which present (15%) of the predictive risk factors), laboratory results (12%), Braden scale (11%), use of medications (10%), age (8%), vital signs (7%), gender (6%), body mass index (3%), length of stay (3%), duration of surgery (3%), critical condition (3%), and other factors that present (19%) of the risk factors. The reviewed papers discussed different domains related to the prediction models of pressure injury, including nursing care, the impact of nursing care on pressure injury, pressure injury, risk factors of pressure injury, biomarkers of pressure injury, machine learning algorithms, and the prediction model of pressure injury. However, although the results obtained from these studies are promising, none of them successfully utilized a fused multi-channel prediction model of pressure injury. We recommend including all pressure injury biomarkers, risk factors, and organizational-related factors in future studies.

## Figures and Tables

**Table 2:** Risk factors and biomarkers of pressure injury.

Type of Variables and References	Summary of Variables
Risk Factors [8,14,43,44,45,46]	Age. Gender. Body mass index. Length of stay. Medications. Vital signs. Anesthesia. Braden scale. Braden subscale (sensory perception, moisture, activity, mobility, nutrition, and friction and shear). Diagnoses include cancer, cardiovascular, diabetes mellitus, renal failure, and respiratory.
Biomarkers [8,44,45,46]	FABP4: Fatty Acid-Binding Protein. IMAT: intramuscular adipose tissue. IL-1α Interleukin-1 Alpha. SEM: sub-epidermal moisture. CK: creatine kinase. H-FAB: heart-type fatty acid binding protein. Mb: myoglobin. Alb: Albumin. Hb: Hemoglobin. CRP: C-Reactive Protein. IL-15: Interleukin-15. TNF-αTumor Necrosis Factor Receptor—Alpha. IFN-α: Interferons—Alpha. GM-CSF: Granulocyte-macrophage colony-stimulating factor.

**Table 3:** Predictive risk factors for pressure injury.

References	Predictive Risk Factors
[22]	Age, female, ASA score, body mass index, Braden score, anemia, respiratory disease, and hypertension.
[23]	Braden score, preoperative fasting blood glucose level, emergency surgery, and types of vasoactive drugs.
[15]	Age, Ethnic, Race, Sex, Admission Source, ASA, LOS at ER, ICU stay, Surgeries, Palliative Orders, history of admissions, need to transitional Unit, steroids, Comorbidity, Depression, PI on admission, Diabetes, Renal Failure, Sepsis, Stroke History, Systolic BP, Diastolic BP, BMIGCS, Weight loss, Saturation, Temperature, Patient Refusal to Change Position, and Skin abnormality on admission, Albumin, BUN, CRP, Creatinine, Hb, MAP, Lactate sodium, Opioids, Steroid Use, Stimuli Anesthesia, Stimuli Paralytics, Stimuli Sedation, Stimuli Tracheostomy, Vasopressor, Artificial Air, Face Mask, Nasal Cannula, Noninvasive Ventilation Pharyngeal, Room Air, Ventilator, and Feeding Tube.
[17]	Three hundred twenty-four features were discussed among the 25 studies.
[24]	Age, gender, diagnosis, cancer, anti-cancer therapy, Waterlow score, laboratory results, medications, length of stay, mechanical ventilation, APACHE score, and blood purification.
[25]	Age, gender, weight, diabetes, vasopressor, isolation, endotracheal tube, ventilator episode, Braden score, and ventilator days.
[26]	Age, gender, admission weight, mean arterial pressure, level of consciousness, medications, diagnoses, and laboratory results.
[14]	Vasopressor medications, temperature, blood pressure, sedation, the severity of illness, oxygenation, and confusion level.
[4]	Sex, age, length of hospital stays, mobility, frictional/shear forces, adrenaline infusion, cardiovascular diseases, disease severity, and nursing workload.
[16]	Demographic factors (Age, BMI, Activity), Treatment Factors (Anesthesia, drugs, Surgeries Duration), and Others (LOS, Hospital Costs).
[27]	Patient history (age and gender), Vital signs (heart rate and blood pressure), lab tests (Hemoglobin and Creatinine levels), LOS, procedures, and medications.
[28]	Age, gender, ethnicity, total intake, total output, length of hospital stays, arterial oxygen saturation, systolic arterial blood pressure, height, daily weight, and glucose (whole blood).
[29]	Reason for admission, clinical laboratory results, patients’ demographics, medical history, and Braden scale.
[30]	Age, gender, diagnoses, diet, pain, paralysis, level of consciousness, skin condition, comorbidities, severity of illness, and department type.
[31]	Age, movement, sensory perception, response, moisture, perfusion, use of medical devices, compulsive position, hypoalbuminemia, HAPI, and surgery.
[32]	Age, gender, diagnosis, Braden score, BMI, heart rate, mean arterial pressure, temperature, laboratory results, and immunocompromised status.
[33]	Oral mucosal, endotracheal tube, vasopressor, albumin, hematocrit, and steroids.
[34]	Age, body mass index, lactate serum, Braden score, vasopressor use, and antifungal medications.
[35]	Duration of surgery, patient weight, duration of the cardiopulmonary bypass procedure, patient age, and disease category.
[36]	GCS, consciousness, gait/transfer, activity, spinal cord injury, albumin, hemoglobin, blood urea nitrogen, chloride, and creatine.
[37]	Albumin, RDW, SAPS-II, CHF, BMI, Glu, Friction/shear score, and Mobility score.
[38]	Laboratory tests, Nursing skin assessment, Surgical time, Vasopressor infusions, and Braden Scale scores.
[39]	Age, gender, diagnoses, length of stay, comorbidities, severity of illness, and Braden scale.
[13]	Gender, age, place of birth, hospital, diagnosis, and APACHE II score.
[40]	Length of anesthesia, wards involved in care, admission reasons, ICU (with/without ventilation), age, sex, and comorbidities.
[41]	Bed Positions, laboratory tests (Creatinine, lactate, pre-albumin, and albumin), Clinical features, admission weight, BMI, activity, and Braden assessment.
[42]	History of PIs, lower care needs mainly mobility, toileting, complex health care, and medication assistance.

**Table 4:** Detail results of machine learning predictive model studies.

Reference	Algorithm	Training %	Testing %	Accuracy %	Sensitivity %	Specificity %	Precision— (PPV)	Recall— (NPV)	AUC
[22]	LR	70	30	NR	40	70.3–94.7	0.73	0.08	NR
[23]	LR	NR	NR	NR	0.63	0.86	NR	NR	0.74
[15]	DT, LR, SVM, LD, RF, MLP, KNN	64–80	20–50	NR	NR	NR	NR	NR	NR
[17]	DT	NR	NR	0.721	0.793	0.721	NR	NR	NR
[24]	LR	NR	NR	83.4	0.66–0.81	0.78–0.96	NR	NR	0.82–0.95
[25]	LR	67	33	0.81	0.65	0.69	0.211	0.956	0.737
[26]	LR, SVM, RF, GBM, NN	80	20	NR	NR	NR	0.12–0.18	0.08–0.70	NR
[14]	RF	67	33	NR	NR	NR	NR	NR	0.79
[4]	DT, LR, RF	NR	NR	NR	0.69–1	0.721–0.99	0.79–0.99	0.82–1	0.876–1
[16]	DT, LR, SVM, NN, RF, Elastic Net	67–90	10–33	63–90	47.8–84.8	70.3–94.7	0.37–0.67	0.61–0.95	NR
[27]	EBM, DT, LR	NR	NR	NR	NR	NR	NR	NR	0.60–0.79
[28]	LR, KNN, RF, BN, SVM	NR	NR	0.84–0.96	NR	NR	0.75–0.94	0.59–0.91	0.77–0.94
[29]	LR, DT, RF	70	30	0.68–0.75	0.39–0.61	0.80–0.91	0.59–0.66	0.74–0.82	0.72–0.82
[30]	LR, RF, SVM, XGBoost	70	30	NR	0.66–0.79	0.69–0.81	0.01–0.02	0.998	0.76–0.82
[31]	LR	83	17	NR	NR	NR	NR	NR	0.87–0.94
[32]	RF	90	10	0.830	NR	NR	NR	NR	0.785–0.792
[33]	Gaussian Naïve Bayes, LR	80	20	A hospital-acquired	0.60–0.85	0.76–0.89	0.86	0.91	0.68–0.82
[34]	Fine-Gray Model	70	30	NR	NR	NR	NR	NR	0.56–0.92
[35]	XGBoost	NR	NR	0.80	0.81	1	1	0.76	0.50–1
[36]	LR, SVM, NN, RF	80	20	0.78–0.91	0.77–0.87	0.79–0.88	NR	NR	0.80–0.94
[37]	LR	67	33	NR	0.69	0.72	NR	NR	0.77
[38]	NN, LR, RF, GB, Adaboost	80	20	NR	NR	NR	NR	NR	0.76–0.81
[39]	LR, RF, NN, DL	70	30	0.86–0.99	0.67–1	0.91–0.99	0.82–0.98	0.88–1	0.71–0.72
[13]	LR, SVM, RF, DT, NN	70	30	0.65–0.68	0.90	0.74	0.08–0.01	0.99–1	0.89
[40]	LR, RF, BART	80	20	0.52–0.55	0.04–0.10	1	0.39–0.58	0.98–0.99	0.89–0.90
[41]	LR, KNN, NB, DT, RF	70	30	0.90–0.97	0.86–0.98	0.87–0.97	0.81–0.96	0.92–0.99	0.90–0.97
[42]	Fine-Gray Model	80	20	NR	NR	NR	NR	NR	0.72–0.75

PPV—Positive Predictive Value; NPV—Negative Predictive Value; AUC—Area Under Curve; NR—Not Reported.

## Data Availability

Not applicable.
